# Disrupted fluid homeostasis in patients with post-Covid-19 syndrome – a case series

**DOI:** 10.3389/fendo.2026.1741517

**Published:** 2026-04-28

**Authors:** Helena Huhmar, Bo C. Bertilson, Olli Polo, Björn Bragée, Per Sjögren

**Affiliations:** 1 Bragée Clinics, Stockholm, Sweden; 2 Department of Neurobiology, Care Sciences and Society, Karolinska Institutet, Huddinge, Sweden

**Keywords:** case series, clinical, fluid homeostasis, osmolality disorders, postCOVID syndrome, Connective tissue disorder

## Abstract

The post-covid-syndrome (PCS) is characterized by severe persisting fatigue and other long haul symptoms following COVID-19 infection. Disease-driving mechanisms are elusive, biomarkers are missing, treatment options few, and yet millions of people worldwide are affected. We hypothesized that the common complaint of polydipsia/polyuria and its attendant effect on osmolality can be quantified and identified as an unrecognized clinical feature of the PCS. We conducted a clinical case-series of 10 consecutive patients with PCS (5 females, 5 males; mean age 44 ± 14 y) who completed questionnaires covering health status, quality-of-life and post-covid symptoms. Detailed anamnestic work-up and clinical assessment were performed, and serum and urine osmolality (S-osm, U-osm) were determined after overnight fasting and fluid deprivation. Polydipsia and/or polyuria were reported by 7 patients, and measures of osmolality were abnormal in all 10 individuals. S-osm was above the reference range (285–292 mOsm/kg) in 9/10 patients (mean 298 ± 4), and U-osm was below the reference (>750 mOsm/kg) in 7/10 patients (mean 707 ± 149). The combination of high serum and low urine osmolality (n=6) was associated with poorer health status, when compared to single abnormality only (n=4). Signs of connective tissue disorders were common among participants, as were previous head-and-neck traumas and activities associated with myofascial distension. Data from this uncontrolled limited sized case series suggests disrupted fluid homeostasis as an unrecognized clinical feature of the PCS. The objective findings of high serum and low urine osmolality are candidates for diagnostic biomarkers as well as potential therapeutic targets and outcome measures.

## Introduction

Following the SARS-CoV-2 (COVID-19) pandemic, a substantial number of individuals worldwide developed long-term post-infectious complications, i.e., post-covid-syndrome (PCS). In PCS, the resolution of the initial infection is followed by debilitating symptoms that, according to the Swedish Health Care system definition, lasts for more than 3 months (1). Afflicted individuals are predominantly women at ages 40–60 years, of whom many experience only mild symptoms during the initial infection (2). Still, more critical symptoms occur as PCS develops, with severe fatigue, dysautonomia and neuro-cognitive problems as dominant features. PCS further leads to impairments in quality-of-life and working ability, with socio-economic consequences (3). Many studies have explored and highlighted potential mechanisms behind PCS (3–5), yet there is currently no consensus explaining the illness. In this clinical case-series, we propose disrupted fluid homeostasis as a previously unrecognized clinical entity of PCS.

Disrupted fluid homeostasis has previously been described in patients suffering from Myalgic Encephalomyelitis/Chronic Fatigue Syndrome (ME/CFS) (6), a condition that shares many signs and symptoms with PCS (4, 7). In that study, concomitant abnormalities of serum and urine osmolalities (S-osm and U-osm) and antidiuretic hormone (ADH) were present, of which the latter is a major regulator of fluid balance. In addition, signs of hypovolemia are frequently described among patients with ME/CFS (6, 8, 9), further denoting the presence of fluid imbalances.

Data on fluid homeostasis in patients with PCS is sparse. A few case-reports have denoted abnormal fluid osmolality and central diabetes insipidus (cDI) in acute and subacute cases of COVID-19 (10–16). However, data on the trajectory of such abnormalities from the acute phase to long-term sequelae are missing. Increased understanding of fluid disruption in PCS may open avenues for appropriate diagnostic procedures and subsequent interventions with the potential to alleviate some of the most debilitating symptoms. As a first step, we investigated fluid homeostasis in a case-series of 10 consecutive patients with PCS.

## Methods

This case-series consists of ten consecutive PCS-patients with neurological symptoms referred by each patient´s family physician to a specialized neurological rehabilitation out-patient clinic. None, except one of the patients had been hospitalized for their initial COVID infection. The patient hospitalized was done so for 4 days in 2021 but without oxygen treatment. All patients visited our rehabilitation clinic between October 2024 and February 2025, and were clinically assessed by a neurologist (HH). The clinical assessment included evaluation of general joint hypermobility, a thorough anamnestic work-up and several PROMs (patient-reported outcomes measure) to capture symptomatology. Aspects of health status and quality-of-life were captured using EQ5D and RAND36, of which the latter is equivalent to SF36. Likert scales of 0–100 yielded estimates of quality-of-life (EQ5D), and physical function (RAND 36), with higher scores reflecting less disability. Of note, a score of 80 or less on the physical functioning scale indicates limitations in physical activities. Cognitive aspects were evaluated using the Mental fatigue Scale (MFS), yielding a composite score of 0-42, with higher values representing worse mental fatigue - a score of 10 was regarded as cutoff for prevalent mental fatigue. Occurrence and severity of typical post-covid symptoms were captured by an internally constructed questionnaire (Bragée Post-Covid Symptom questionnaire, BPCS). The BPCS form was adapted from the previously published “COVID-19 Yorkshire Rehabilitation Scale” (17), and included certain symptoms not included in the original questionnaire and further restructured for comprehensiveness (see Appendix 1). The severity of each symptom was captured on a 0–7 Likert scale, with 7 being the worst. Furthermore, working ability was captured on a scale 0-100% in which 100 reflects full working capacity.

After an overnight fasting and ten hours long fluid deprivation, patients delivered morning samples of blood and urine to the Karolinska University Laboratory for measurements of blood glucose, serum sodium, potassium, and osmolality, as well as urine osmolality. Medical charts were reviewed to collect historical data on sodium and glucose to estimate pre-COVID measures of S-osm by using the classical algorithm (2*Na) + Glucose + Urea (all three in mmol/L). However, measures of circulating urea were unavailable in our participants, and a parsimonious algorithm (without urea) was applied as a consequence. Medical charts were further used to collect concomitant medication that may influence thirst, diuresis and electrolyte handling.

All participants gave their written informed consent prior to inclusion, allowing for a retrospective evaluation of their clinical examinations. Ethical approval was granted by the Swedish Ethical Review Authority (2024-07116-01).

*Statistical analysis*. MS-Excel (Microsoft, Redmond, Washington) and Statistics Kingdom (statskingdom.com, an on-line statistical resource, Melbourne, Australia) were used for data processing. For ease of understanding, all data are presented as mean (SD), but statistical analyses used parametric tests for normally distributed variables and non-parametric tests for non-normally distributed variables.

## Results

**Figure 1:**
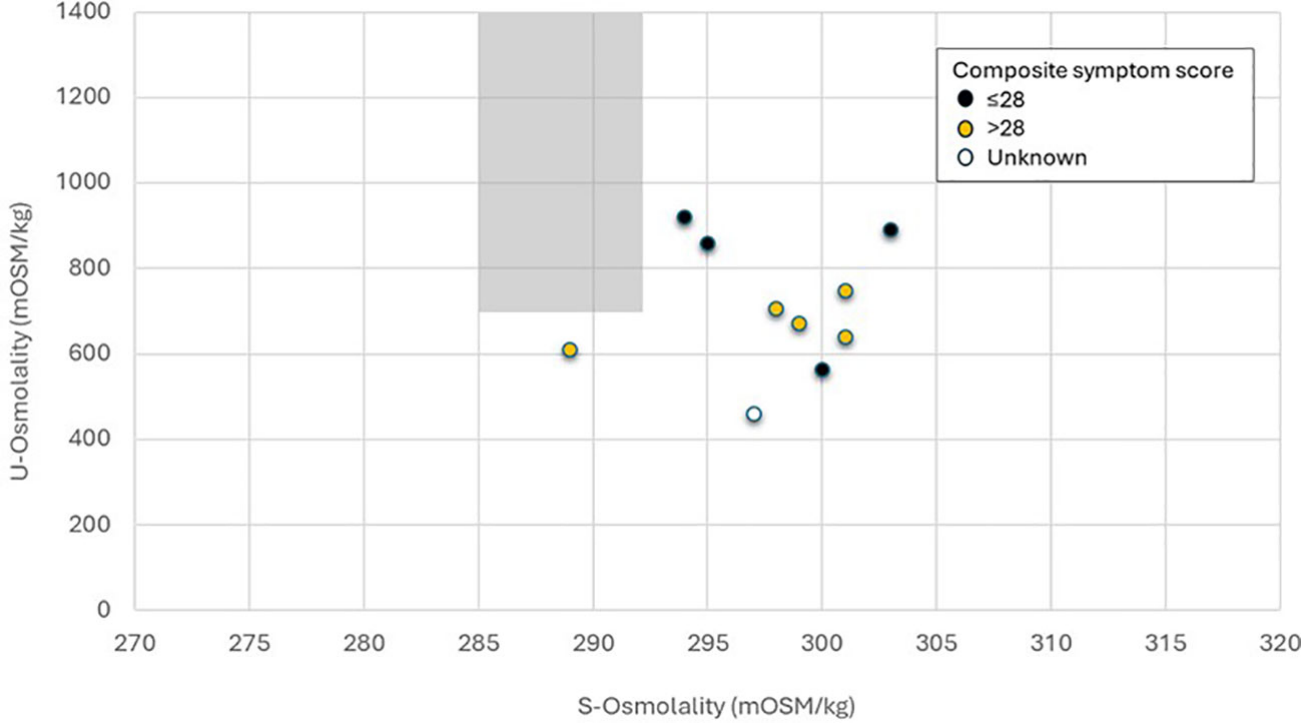
U-Osm in relation to S-Osm in 10 patients with post-covid following 10-hour fluid deprivation. Each dot represents one individual, coloured according to self-reported symptom score in relation to the median (28) of the group, with yellow-filled circles reflecting more severe symptoms. The Composite symptom score could range from 0-49 (with 49 as maximum severity) and was calculated from the sum of the 7 symptoms included in Tab 1, i.e., fatigue, headache, heart palpitations, dizziness, sleep disturbances, concentration difficulties and memory issues, reported on a 0–7 scale respectively. One patient did not report individual symptoms (Unknown). Shaded area represents the reference ranges for S-Osm (285–292 mOSM/kg) and U-Osm (>750 mOSM/kg.

The characteristics of the 10 consecutive patients with PCS included in this case-series are presented in **Table 1**, with a mean age of 44 ± 14 years, and half (50%) being female. All patients reported an initial COVID infection before 2023 with sustained post-infectious complications lasting >1 year, ranging from 1 to 5.5 years. Mean measures of blood pressure, heart rate, as well as serum sodium and potassium levels were all within the normal range. Mean fasting blood glucose were slightly elevated in the group (6.2 ± 2.1 mmol/L), which was driven by one hyperglycemic participant (12.2 mmol/L). Excluding that individual yielded a mean blood glucose of 5.6 ± 0.3 mmol/L (n=9). General joint hypermobility was present in 50% of the patients and another 20% were close to fulfill the criteria. Polydipsia and/or polyuria was reported in 70% of the patients. The mean measures of osmolality diverged from normal values in both serum (298 ± 4; reference range 285–292 mOsm/kg) and urine (707 ± 149, reference value >750 mOsm/kg). Individual levels of serum and urine osmolality are given in **Figure 1**, revealing that all individuals deviated from normal physiologically (shaded area). Of note, osmolality measures remained abnormal for all individuals also after taking blood glucose levels into consideration, according to established literature in medical physiology (18). We also collected historical laboratory data on sodium and glucose to estimate pre-COVID levels of serum osmolality. As presented in **Table 2**, no deviations from normal osmolality were detected for calculated pre-COVID levels (before 2020), albeit without urea values. The calculated osmolality at the time-point for the current examinations (2024-25) deviated from the actual measurements of S-osm, being consistently lower with a mean difference of 9.4 mOsm/kg ranging from 3.8-13.1 mOsm/kg. Of note, normal values of S-urea ranges between 2.5-to-7.8 mmol/L, indicating remaining deviations compared to analytically derived measures, if normal S-urea values were added to the algorithm.

**Table 1:** Demographic data of patients with post-covid included in the case-series.

Characteristics	N	Values
Female, n (%)	n=10	5 (50)
Age in years, mean (SD)	n=10	44 (14)
Systolic blood pressure, mean (SD)	n=8	124 (10)
Diastolic blood pressure, mean (SD)	n=8	84 (7)
Heart rate, mean (SD)	n=7	64 (6)
General joint hypermobility, n (%)	n=10	5 (50)
Polyuria or polydypsia, n (%)	n=10	7 (70)
Quality-of-life, 0–100 mean (SD)	n=9	36 (18)
Physical function, 0–100 mean (SD)	n=10	46 (23)
Mental fatigue, 0–42 mean (SD)	n=10	22 (7)
Working ability, 0-100% mean (SD)	n=9	26 (27)
Composite symptom score, 0–49 mean (SD)	n=9	25 (15)
Fatigue, 0–7 mean (SD)	n=9	6.2 (2.0)
Headache, 0–7 mean (SD)	n=9	3.9 (2.4)
Heart palpitations, 0–7 mean (SD)	n=9	3.2 (2.8)
Dizziness, 0–7 mean (SD)	n=9	4.7 (2.3)
Sleep disturbances, 0–7 mean (SD)	n=9	4.9 (1.5)
Concentration difficulties, 0–7 mean (SD)	n=9	4.8 (2.1)
Memory difficulties, 0–7 mean (SD)	n=9	4.4 (1.7)
fB-Glucose in mM, mean (SD) [Ref range 4.2-6.0]	n=10	6.2 (2.1)
s-Na in mM, mean (SD) [Ref range 137-145]	n=10	141 (1)
s-K in mM, mean (SD) [Ref range 3.5-4.6]	n=9	4.0 (0.3)
Osmolality (mOsm/kg)		
Serum, mean (SD) [Ref range 285-292]	n=10	298 (4)
Urine, mean (SD) [Ref range >750]	n=10	707 (149)

All data are presented as mean (SD) for ease of understanding or n (%). General joint hypermobility was determined by using the Beigthon score and the 5PQ, as described (ref). Quality-of-life was assessd by the EQ5D; physical function was derived from the domain for physical functioning in RAND36; mental fatigue was derived from the Mental Fatigue Scale; and the Composite symptom score represents the sum of the individual symptoms (fatigue, headache, heart palpitations, dizziness, sleep disturbances, concentration difficulties and memory issues) reported on a Likert scale 0–7 with 7 as worst possible severity. fB-glucose represents fasting blood glucose levels, s-Na and s-K reflects serum values of sodium and potassium, respectively.

**Table 2:** Calculated and measured serum osmolality, and concomitant medications with potential impact on osmolality, presented for each individual included in this case-series (n=10).

ID	Gender	Age	S-Osm (mOsm/kg) Calculated^a^			S-Osm (mOsm/kg) Measured	Medications With potential osmolytic impact at the time of sampling^c^
	Male/female	Yrs.	<2020	2020-24	2024-25^b^	2024-25^b^	
Pat 01	M	58	286	286	288	301	No
Pat 02	M	50	283	289	288	298	No
Pat 03	M	51	NA	NA	283	294	No
Pat 04	F	26	283	288	287	295	SNRI, since 2019
Pat 05	F	60	280	284	288	301	Corticosteroids, non-systemically administrated Macrogol, taken pre-colonoscopy >2w before osmolytic sampling
Pat 06	F	29	288	285	285	289	Corticosteroids, non-systemically administrated
Pat 07	M	42	291	294	298	303	Diuretics, since 2022
Pat 08	Trans-man	32	285	289	289	299	No
Pat 09	F	30	283	285	287	300	SSRI, since 2023
Pat 10	F	64	284	289	288	297	SNRI, since 2024

^a^ Osmolality calculated from Sodium (mM)*2 + Glucose (mM), derived from electronic medical charts.

^b^ Time point of current clinical assessment and biosampling.

^c^ Including Lithium, diuretics, SGLT2-inhibitors, SSRI, SNRI, antipsychotics, desmopressin, corticosteroids, and substances with a direct osmolytic effect.

SSRI, Selective serotonin reuptake inhibitor.

SNRI, Serotonin–norepinephrine reuptake inhibitor.

Concomitant medication are presented in **Table 2**, with one patient being on diuretics, another on SSRI and two on SNRI. Macrogol, with strong osmolytic impact, was taken by one patient in connection with colonoscopy, performed >2 week prior to the current examinations. Two patients were on corticosteroid treatment, one using skin cream and the other inhalation, i.e., both non-systematically administered. No patients were on lithium, SGLT2-inhibitors, antipsychotics or desmopressin.

Self-reported quality-of-life was low (mean 36 on a 0–100 scale) among our participants with prevalent mental fatigue and impaired physical functioning (Tab 1). Individual symptoms reported by the patients revealed a high severity of fatigue (mean 6.2 ± 2.0 on a 0–7 scale) and reasonably high severity of dysautonomia (heart palpitation, dizziness, sleep disturbance) and neurocognitive (memory and concentration difficulties) symptoms, with mean values between 3.2 to 4.9 on a 0–7 scale. The average working ability reported by the group was 26% and all individuals except 2 evaluated their working ability to less than 25%. One individual reported 90% working ability.

Group comparison based on osmolality measures indicated a poorer health state among individuals with abnormal measures for serum and urine osmolality in combination (n=6), as compared to those with only one (n=4). Individuals with abnormal measures for osmolality in combination had numerically lower physical functioning (mean 34, range 5–70 vs. mean 63, range 50-75) captured by RAND-36 (with 100 reflecting no limitations), and a higher symptom severity score (mean 37, range 28–46 vs. mean 24, range 14-41) based on the composite symptom score (Tab 1) with a score of 49 representing worst possible symptoms. Statistical tests were prohibited due to limited sample size.

Finally, the detailed anamnestic work-up performed during the clinical assessment revealed a frequent occurrence of physical traumas and/or activities related to myofascial distension (such as yoga, chiropractic manipulation and advanced dancing) among our patients (**Table 3**). Head-and-neck traumas were part of the medical history in 5 out of 10 patients (50%) and activities associated with myofascial distension were present in 9 out of 10 patients (90%). One patient reported no previous physical traumas or activities including myofascial distension, but had severe spinal degeneration in cervical segments, according to radiological examinations.

**Table 3:** The presence of hypermobility as well as anammenstically derived head/neck traumas, and activities with myofascial distension for each individual included in this case-series (n=10).

ID	Gender	Age	Hypermobility	Head and/or Neck trauma	Activities with myofascial distension^a^
	Male/female	Yrs.	Yes/Partly/No	Description	Description
Pat 01	M	58	N	None	>20 visits to a naprapath with adjustments/manipulations of the spine.
Pat 02	M	50	Y	None	>6 years of troop gymnastics in his youth; >15 visits to a chiropractor with adjustments/manipulations of the spine.
Pat 03	M	51	Y	None	Elite gymnast in youth with >20 years of gymnastic exercise several times per week; >50 visits to a chiropractor with adjustments/manipulations of the spine.
Pat 04	F	26	N	None	>6 years of ballet dancing in her youth; From 17 y of age practiced yoga regularly
Pat 05	F	60	Y	Bicycle accident at age 45 and car accident at age 47, both with severe head/neck trauma.	Practiced yoga on a daily basis for ~20 years
Pat 06	F	29	Partly	Head/neck trauma on 3 occasions: twice falling of a horse (at age 11-14) and once at age 40 when a platform collapsed (falling 4m).	Regular therapeutic manipulation of the spine over a 2-year period starting at ~20 years of age.
Pat 07	M	42	Partly	Three times hit by car, twice being on a bike, once as a pedestrian. Physically assaulted twice, at 15 and 34 years of age, respectively.	>20 visits to a chiropractor when 22-to-25 years of age with adjustments/manipulations of the spine.
Pat 08	Trans-man	32	Y	Several sports related accidents with 3–5 concussions as adolescent.	Practiced Yin Yoga on a regular basis since age 26 y
Pat 09	F	30	Y	During adolescence involved in three accidents with head/neck trauma (twice landing on the head doing gymnastics, once part of a bus accident).	Dancing and gymnastics on a regular basis between 6–19 years of age. Attending ballet school between 15–19 years of age with 4–5 hours of practicing every weekday + weekend dancing.
Pat 10	F	64	N	*No explicit trauma reported. Radiologically determined spinal degeneration present.	None

^a^ Myofascial distension on a regular basis prior to post-covid development, derived from anamnestic history for each patient, including activities like chiropractic manipulation or similar, extensive yoga or dance practicing.

## Discussion

Our findings, derived from a clinical case-series, suggest dysregulated fluid homeostasis as an unrecognized clinical entity of PCS. A majority of the included patients had symptoms of polydipsia/polyuria and all of them exhibited abnormal measures of osmolality in either serum, urine or both. Furthermore, connective tissue abnormalities, history of physical traumas, and spare-time activities including myofascial distension were frequent. Our findings may promote research on how Covid-19 affects the hormonal control of fluid balance and also the role of connective tissue abnormalities.

Studies on fluid balance in relation to PCS are sparse, yet several studies have described signs of disturbed fluid balance in conditions with post-covid-resembling symptoms (6, 19). For example, hypovolemia is common among patients suffering from ME/CFS (8, 9), and in a clinical setting we showed that intermittent saline-infusion improved symptoms of dysautonomia, quality-of-life and working ability in such patients (20). Furthermore, we found abnormally low levels of ADH in conjunction with disrupted fluid homeostasis in patients with ME/CFS (6), which also has been described in other long-term fatigue conditions (19). Several recent case reports have described the same phenomenon in more acute conditions, as a sequela of Covid 19 infection (10–16). Those case reports describe individuals who developed severe polyuria, excessive thirst and nocturia following SARS-CoV-2 infection, on average 3–5 weeks after a positive Covid PCR test. Measures of osmolality were abnormal, ADH levels undetectable (if quantified), and cases were diagnosed with cDI. Importantly, all subjects improved substantially when prescribed the ADH-analogue desmopressin, supporting the role of dysfunctional ADH secretion in this scenario. Neither malignancy nor hemorrhages explained the sudden cDI in any of the cases and signs of hypophysitis were found in only one of them (12).

Downregulated ADH secretion is a strong candidate explaining deviations from normal osmolality in patients with acute Covid, PCS, ME/CFS, or other forms of chronic fatigue. The mechanisms behind the disrupted ADH secretion remain elusive and may include both direct and indirect damage to ADH secreting cells. ACE-2 receptors, acting as gateway for SARS-CoV-2 infiltration, are abundantly expressed in hypothalamus and pituitary tissue (21), and autopsy studies suggest that the brainstem could be a major target of SARS-CoV-2 in the CNS (22).

An interesting observation in our cohort is the high prevalence of patients with hypermobility and/or activities related to myofascial distension. Individuals with connective tissue disorders are clearly overrepresented in this patient group (23), and may be particularly vulnerable to the infiltration of viruses to culprit areas with subsequent biological impact. Whether the frequent occurrence of physical traumas and/or activities related to myofascial distension are of importance in the development of PCS remains undefined. However, we have detected (through detailed anamnestic and clinical procedures) a high occurrence of myofascial distension in patients suffering from ME/CFS. Future studies are needed to help elucidate any potential role of myofascial distension, with or without underlying connective tissue disorder, in disease genesis.

Limitations of this study include the fact that this is a limited sized case-series of patients with PCS. A small sample size is prone to bias, and it is uncertain whether our ten consecutive patients are representative of patients with PCS. Furthermore, data on ADH levels as well as circulating urea would have contributed with important insight to our conclusions. Measures of circulating urea would have improved the accuracy for calculated osmolality, and whether urea concentrations is elevated in this patient group remains to be shown. Finally, we were unable to fully control for all circumstances that may induce hyperosmolality, although our review of the medical charts excluded concomitant medication as a general explanation to changes in osmolality.

In conclusion, data derived from this uncontrolled limited sized case series suggests disrupted fluid homeostasis as an unrecognized entity that may have diagnostic value and could potentially be target for treatment in PCS. We are foreseeing replications of our findings in more large scale studies, taking into account ADH levels and potential determinants of disturbed osmolality to further understand and combat disrupted fluid homeostasis in this patient group.

## Data Availability

The raw data supporting the conclusions of this article will be made available by the authors, without undue reservation.
