# Supplementary material for: Disrupted fluid homeostasis in patients with post-Covid-19 syndrome – a case series
Source: Front Endocrinol (Lausanne). 2026 Apr 28;17:1741517. doi: 10.3389/fendo.2026.1741517 (PMC13160803; doi:10.3389/fendo.2026.1741517)
Supplement: Supplementary file 1 [file DataSheet1.pdf]

Appendix 1

The post-covid symptom questionnaire used in this study. Original language Swedish, translated to English.

Bragée Post-Covid Symptom Questionnaire (BPCS-01)_before rehab

Name: **Pers.id:**

Date:

The purpose of this questionnaire is to capture in detail the problems you are experiencing after having COVID infection. Your answers will be recorded in your medical record and will be used to evaluate treatment and any further interventions. Please allow 10–15 minutes to complete the questionnaire.

Please answer the questions as accurately as you can, thank you.

'Now' refers to how you are feeling today/last week.

"Pre-COVID" refers to how you were feeling before you got the infection, as best you can remember.
Rate your symptoms when you are feeling at their worst. Circle 0 for no symptoms and 7 if being worst possible

0 1 2 3 4 5 6 7
No symptom Worst possible

**1. Shortness of breath**	a) At rest	Now	0	1	2	3	4	5	6	7
		Pre COVID	0	1	2	3	4	5	6	7
	b) When changing position, like getting dressed	Now	0	1	2	3	4	5	6	7
		Pre COVID	0	1	2	3	4	5	6	7
	c) Walking up stairs	Now	0	1	2	3	4	5	6	7
		Pre COVID	0	1	2	3	4	5	6	7
**2. Throat problems**	a) Feeling of infection and/or coughing	Now	0	1	2	3	4	5	6	7
		Pre COVID	0	1	2	3	4	5	6	7
	b) Voice change	Now	0	1	2	3	4	5	6	7
		Pre COVID	0	1	2	3	4	5	6	7
**3. Fatigue/exhausti on**	- Fatigue that does not improve with rest	Now	0	1	2	3	4	5	6	7
		Pre COVID	0	1	2	3	4	5	6	7
**4. Smell/taste**	- Altered sense of smell	Now	0	1	2	3	4	5	6	7
		Pre COVID	0	1	2	3	4	5	6	7
	- Altered sense of taste	Now	0	1	2	3	4	5	6	7
		Pre COVID	0	1	2	3	4	5	6	7
**5. Vision impairment**	- Experiencing blurred vision	Now	0	1	2	3	4	5	6	7
		Pre COVID	0	1	2	3	4	5	6	7
	- Sensitive to visual stimuli, e.g. light or movement	Now	0	1	2	3	4	5	6	7
		Pre COVID	0	1	2	3	4	5	6	7
**6. Pain/discomfort**	- Chest pain	Now	0	1	2	3	4	5	6	7
		Pre COVID	0	1	2	3	4	5	6	7

	- Joint pain	Now<br>Pre COVID	0<br>0	1<br>1	2<br>2	3<br>3	4<br>4	5<br>5	6<br>6	7<br>7
	- Muscle pain	Now<br>Pre COVID	0<br>0	1<br>1	2<br>2	3<br>3	4<br>4	5<br>5	6<br>6	7<br>7
	- Headache	Now<br>Pre COVID	0<br>0	1<br>1	2<br>2	3<br>3	4<br>4	5<br>5	6<br>6	7<br>7
	- Abdominal pain	Now<br>Pre COVID	0<br>0	1<br>1	2<br>2	3<br>3	4<br>4	5<br>5	6<br>6	7<br>7
**7. Dysautonomia/ impact on the autonomic nervous system**	- Heart palpitations	Now<br>Pre COVID	0<br>0	1<br>1	2<br>2	3<br>3	4<br>4	5<br>5	6<br>6	7<br>7
	- Dizziness	Now<br>Pre COVID	0<br>0	1<br>1	2<br>2	3<br>3	4<br>4	5<br>5	6<br>6	7<br>7
	- Discomfort when standing up	Now<br>Pre COVID	0<br>0	1<br>1	2<br>2	3<br>3	4<br>4	5<br>5	6<br>6	7<br>7
	- - Balance problems	Now<br>Pre COVID	0<br>0	1<br>1	2<br>2	3<br>3	4<br>4	5<br>5	6<br>6	7<br>7
	- Frequent urination/thirsty	Now<br>Pre COVID	0<br>0	1<br>1	2<br>2	3<br>3	4<br>4	5<br>5	6<br>6	7<br>7
	- Sleep disturbance, e.g. difficulty falling asleep or waking up at night	Now<br>Pre COVID	0<br>0	1<br>1	2<br>2	3<br>3	4<br>4	5<br>5	6<br>6	7<br>7
**8. Exercise-induced symptom worsening, so-called PEM-PESE.**	- Significantly worse/completely knocked out for a longer period of time after physical, cognitive or emotional strain	Now<br>Pre COVID	0<br>0	1<br>1	2<br>2	3<br>3	4<br>4	5<br>5	6<br>6	7<br>7
**9. Cognition**	- Concentration difficulties	Now<br>Pre COVID	0<br>0	1<br>1	2<br>2	3<br>3	4<br>4	5<br>5	6<br>6	7<br>7
	- Memory problems	Now<br>Pre COVID	0<br>0	1<br>1	2<br>2	3<br>3	4<br>4	5<br>5	6<br>6	7<br>7
	- Difficult to plan	Now<br>Pre COVID	0<br>0	1<br>1	2<br>2	3<br>3	4<br>4	5<br>5	6<br>6	7<br>7
	- Difficult to find words	Now<br>Pre COVID	0<br>0	1<br>1	2<br>2	3<br>3	4<br>4	5<br>5	6<br>6	7<br>7

**9. Mood**	- Feeling worried	Now<br>Pre COVID	0<br>0	1<br>1	2<br>2	3<br>3	4<br>4	5<br>5	6<br>6	7<br>7
	- Feeling down	Now<br>Pre COVID	0<br>0	1<br>1	2<br>2	3<br>3	4<br>4	5<br>5	6<br>6	7<br>7
	- Have intrusive unwanted memories of past trauma	Now<br>Pre COVID	0<br>0	1<br>1	2<br>2	3<br>3	4<br>4	5<br>5	6<br>6	7<br>7
**10. Other symptoms**	- Nausea	Now<br>Pre COVID	0<br>0	1<br>1	2<br>2	3<br>3	4<br>4	5<br>5	6<br>6	7<br>7
	- Skin rash	Now<br>Pre COVID	0<br>0	1<br>1	2<br>2	3<br>3	4<br>4	5<br>5	6<br>6	7<br>7
	- Skin irritation such as itching, tingling, or numbness	Now<br>Pre COVID	0<br>0	1<br>1	2<br>2	3<br>3	4<br>4	5<br>5	6<br>6	7<br>7
	……………………………..<br>…………………. *(state)*	Now<br>Pre COVID	0<br>0	1<br>1	2<br>2	3<br>3	4<br>4	5<br>5	6<br>6	7<br>7
	……………………………..<br>…………………. *(state)*	Now<br>Pre COVID	0<br>0	1<br>1	2<br>2	3<br>3	4<br>4	5<br>5	6<br>6	7<br>7
**11. Working ability**	Estimate your work capacity in %, before and after COVID.<br>0% is no work capacity<br>100% is full work capacity	Now<br>Pre COVID	I----I----I----I----I----I----I----I----I----I----I<br>0 50 100<br>I----I----I----I----I----I----I----I----I----I----I<br>0 50 100							
